# Sedative effect and safety of different doses of S-ketamine in combination with propofol during gastro-duodenoscopy in school-aged children: a prospective, randomized study

**DOI:** 10.1186/s12871-022-01885-1

**Published:** 2022-11-12

**Authors:** Junxia Wang, Weidong Hu, Xianliang Zhao, Weilian Ren, Xin Huang, Bin Zhang

**Affiliations:** 1 Department of Pediatrics, The First Affiliated Hospital of Shandong First Medical University & Shandong Provincial Qianfoshang Hospital, Jinan, 250012 China; 2 Department of Anesthesiology, Children’s Hospital Affiliated to Shandong University & Jinan Children’s Hospital, No. 23976, Jingshi Road, Jinan, 250012 China; 3 School of Public Health, Cheeloo College of Medicine, Shandong University, Jinan, 250012 China

**Keywords:** Child, Gastro-duodenoscopy, S-ketamine, Propofol

## Abstract

**Background:**

Propofol combined with opioids can reduce the dosage of propofol and improve the safety of endoscopy. However, there are few studies on propofol combined with S-ketamine in children undergoing gastro-duodenoscopy. We aim to determine the sedative effect and safety of different doses of S-ketamine in combination with propofol in school-aged children undergoing gastro-duodenoscopy.

**Methods:**

This is a prospective, randomized trial. Totally, 120 school-aged children who underwent gastro-duodenoscopy were randomly allocated into Group P, Group S_0.3_, Group S_0.5_ and Group S_0.7_. During induction, children in Group P, Group S_0.3_, Group S_0.5_ and Group S_0.7_ received 0, 0.3 mg.kg^−1^, 0.5 mg.kg^−1^ and 0.7 mg.kg^−1^ S-ketamine, respectively, following 3 mg.kg^−1^ propofol injection. During gastro-duodenoscopy, 1 mg.kg^−1^ of propofol was added according to the condition of the children and the BIS (bispectral index) value. The primary outcome was smooth placement rate of the first endoscope insertion. The secondary outcome was the times of additional propofol, the total amount of propofol, adverse events, recovery time, length of PACU (post anesthesia care unit) stay and endoscopist satisfaction.

**Results:**

The smooth placement rate of the first endoscope insertion in Group P, Group S_0.3_ and Group S_0.5_ was significantly lower than that in Group S_0.7_ (16.70%, 34.50%, 50.00% vs. 83.30%, respectively, *P* < 0.001). The times of additional propofol in Group S_0.3_ (*P* = 0.018), Group S_0.5_ (*P* = 0.014) and Group S_0.7_ (*P* = 0.001) were significantly less than Group P. The total amount of propofol in Group S_0.7_ was significantly less than Group P (*P* < 0.001). The incidence of intraoperative hypotension in Group S_0.5_ and Group S_0.7_ was low. Group S_0.7_ had significantly higher incidence of postoperative dizziness (*P* = 0.003), longer PACU stay (*P* = 0.018) and higher endoscopist satisfaction (*P* = 0.001) than Group P. There was no difference in the recovery time among groups.

**Conclusion:**

S-ketamine (0.7 mg.kg^−1^) in combination with propofol can provide satisfactory sedative effect and reduce the dosage of propofol in school-aged children undergoing gastro-duodenoscopy, but there are higher incidence of postoperative dizziness and longer PACU stay.

## Background

Deep sedation during gastro-duodenoscopy can help pediatric patients to tolerate endoscopy examination painlessly and comfortably and can also facilitate the completion of operation by endoscopists (Chung and Lightdale, [5]). However, adverse events caused by sedative drugs are potential risk factors for pediatric patients undergoing gastrointestinal endoscopy. Currently, the optimal sedative or drug combination regimens for sedation in children undergoing gastrointestinal endoscopy are lacking (Akbulut, et al., [1]).

Propofol is a sedative with advantages of ease of titration, rapid onset of action and brief duration of effect, which is currently considered as the most common sedative for gastro-duodenoscopy in children (Alletag, et al., [3], Chung and Lightdale, [5]). However, propofol administration can cause dose-dependent adverse events. The incidence of respiratory and circulatory complications can be as high as 23.4% in children sedated with propofol alone (Narula, et al., [18]). Ketamine, an ideal induction drug and a common adjuvant for sedation during gastro-duodenoscopy, has positive sympathomimetic effects and can maintain hemodynamic stability and airway reflexes (Barrett, et al., [4]). Compared with opioids, sedation by ketamine combined with propofol has decreased rates of respiratory adverse effects and circulatory complications (Yan, et al., [24], Jalili, et al., [14]), without increasing the recovery time (Eberl, et al., [6]). In addition, ketamine does not inhibit μ receptors, thus avoiding gastrointestinal obstruction, and does not affect gastrointestinal function (Erstad and Patanwala, [8]). Therefore, ketamine is more suitable for sedation in patients with gastrointestinal disorders.

S-ketamine, an S + isomer of ketamine, is twice as potent as ketamine, which can achieve more reliable sedation and analgesia with a relatively low risk of side effects. S-ketamine offers many advantages as an adjuvant, including maintenance of airway tone and hemodynamic stability, and serves as an ideal choice for anesthesia and sedation (Trimmel, et al., [22]). Hypersalivation induced by ketamine can be relieved by concomitant administration of anticholinergic drugs (Peltoniemi, et al., [19]). However, some side effects of S-ketamine, such as induction of vomiting and psychotomimetic effects in children, limit its single administration for sedation (Nakao, et al., [17]). Propofol has anti-anxiety properties that can inhibit ketamine-induced psychiatric symptoms (Friedberg, [10]), and has intrinsic antiemetic properties that can reduce the risk of nausea (Yan, et al., [24]). Therefore, propofol combined with ketamine/S-ketamine is recommended as a kind of administration mode for sedatives (Eich, et al., [7], Smischney, et al., [20]). It has been shown that different doses of ketamine lead to differences in propofol dosage and sedative effects during gastro-duodenoscopy in children (Hayes, et al., [13]). At present, there is rare data on the sedative effect and safety of different doses of S-ketamine in combination with propofol for gastro-duodenoscopy in children.

Therefore, in this prospective study, we investigated the sedative effect and safety of intravenous administration of different doses of S-ketamine in combination with propofol for gastro-duodenoscopy in school-aged children.

## Methods

### Ethics

The study was approved by the Ethics Review Board of Children’s Hospital Affiliated to Shandong University (ethic code: ETYY-2020219). All methods were performed in accordance with the relevant guidelines and regulations under the committee supervision. Written informed consents were obtained from the legal guardians of all children.

### Design

This is a prospective, observer-blinded, randomized trial conducted in Children’s Hospital Affiliated to Shandong University (Jinan, China). This trial was registered at www.clinicaltrials.gov (ChiCTR2100044321; Date: 16/03/2021).

### Study participants

School-aged children (6 to 12 years old) with ASA I or II and undergoing gastro-duodenoscopy were enrolled. Exclusion criteria: subjects with upper respiratory tract infection, active gastrointestinal bleeding, nausea, vomiting, mental disorder, obesity (Body Mass Index > 35 kg.m^−2^) or any contraindication to study medications were excluded.

### Randomization and masking

All enrolled children were allocated sequentially into Group P, Group S_0.3_, Group S_0.5_ and Group S_0.7_ using a double blinded randomization system at a 1:1:1:1 ratio. The randomized block 4 design by a random number generating computer software was used for randomization. According to a previous study (Peltoniemi, et al., [19]) and our clinical experience, and to determine the dose dependent effect of S-ketamine, the doses of S-ketamine were determined at 0, 0.3, 0.5, and 0.7 mg.kg^−1^. Then, S-ketamine (0, 0.3, 0.5, or 0.7 mg.kg^−1^) were administrated intravenously to Group P, Group S_0.3_, Group S_0.5_ and Group S_0.7_, respectively. Participants and their parents, the anesthesiologists, the endoscopists, and the nurses of PACU (post anesthesia care unit) were blinded to patient allocation.

### Procedures

A standardized anesthetic regimen was used in all subjects. An intravenous catheter was placed before the procedure. At 10 min before induction, 5 ml of 2% lidocaine cement (Henan Kangyuan Bioengineering Technology Co., Ltd., Xinyang, China) was administered orally. The patient was placed in the lateral recumbent position with the head slightly tilted back to open the airway. Oxygen was inhaled through nasal prongs (FiO2 100%, and oxygen flow 1L/min) to maintain oxygen supply. Electrocardiogram, pulse oximetry, and non-invasive blood pressure cuff were applied. The end-expiratory carbon dioxide catheter of the anesthesia machine was connected to the nostril of patient to continuously monitor breathing. The bispectral index (BIS) value measured by bispectral index sensor (Covidien, Mansfield, MA) was used to continuously monitor depth of anesthesia.

An unblinded study investigator, who did not participate in patient care during the sedation and endoscopy, prepared the S-ketamine injection (Jiangsu Hengrui Medicine Co., Ltd., Lianyungang, China). According to random assignment serial numbers, the 0, 0.3, 0.5 and 0.7 mg.kg^−1^ of S-ketamine was respectively extracted to a 10 ml syringe and then diluted to 10 ml of volume with normal saline. For Group P, 10 ml of normal saline was used. The prepared S-ketamine solution was transferred to an anesthesiologist, who was blinded to patient allocation and was responsible for sedation management. During the induction of anesthesia, glycopyrrolate (5 μg.kg^−1^; Lisite Ltd., Chengdu, China) was administered intravenously first to reduce the salivation caused by S-ketamine and gastro-duodenoscopy, and then lidocaine (0.05 mg.kg^−1^; Tiancheng Ltd., Shijiazhuang, China) was intravenously administered to reduce the pain of propofol injection. Then, propofol (3 mg.kg^−1^; Fresenius Kabi Canada Ltd., Toronto, ON, Canada) was slowly injected (over 1 min). The breathing was closely monitored during the injection process. The injection should be stopped if there is apnea, slow breathing or SpO2 drop; and, if the symptoms are relieved by jaw thrust or mask ventilation, the injection can be continued. Following propofol administration, the prepared S-ketamine was slowly injected (over 30 s). Propofol was administered earlier than S-ketamine in order to prevent the identification of different sedative effects between Group P and other groups by blinded anesthesiologists. All patients were administrated the standard sedation of 3 mg.kg^−1^ propofol + 10 ml study medication. Gastro-duodenoscopy was performed by endoscopists with more than 5 years of working experience about 1 min after S-ketamine administration. During the gastro-duodenoscopy, if there is physical movement, choking or hiccups, heart rate increase over 20% of before endoscope insertion, or BIS value greater than 85, propofol (1 mg.kg^−1^) will be added. After gastro-duodenoscopy, the patients were transferred to PACU. When the Aldrete score reached 10 and there were no adverse reactions, they were transferred out of PACU.

### Study outcomes

The data collection was conducted by anesthesiologists and the blinded nurses from PACU. The heart rate and mean arterial pressure were recorded before induction, at 1 min after induction, and at 5 min intervals during the procedure. The BIS value was recorded every 5 min.

The primary outcome was the smooth placement rate of first endoscope insertion. If there is no physical movement, coughing, gagging or airway obstruction, and the increase in heart rate is less than 20% of the pre-endoscope insertion during insertion, smooth placement of endoscope was defined.

The secondary outcomes included the times of additional propofol, the total amount of propofol, adverse events, recovery time, length of PACU stay and endoscopist satisfaction. Adverse reactions after induction and during gastro-duodenoscopy included hypoxemia (SpO2 < 90% and more than 1 min), hypotension (mean arterial pressure lower than 20% of pre-induction), bradycardia (heart rate less than 60 beats per min), coughing and hiccups. Adverse reactions during the recovery including headache, dizziness, vomiting, nausea, visual disturbance (blurred or double-vision), and hallucinations. The endoscopist satisfaction was evaluated using a ten-point scale (1–3: unsatisfactory; 4–6: average satisfaction; 7–10: satisfactory) (Akbulut, et al., [1]).

### Sample size calculation

We used smooth placement rate of first endoscope insertion as the primary outcome. Based on the results of our pilot study, the smooth placement rate of first endoscope insertion after single injection of propofol was 20%. We anticipated a 35% increase in the smooth placement rate after combined injection of propofol and S-ketamine. Assuming an alpha error of 0.05 and a power of 80%, we calculated that at least 27 patients per group were required to detect a statistically significant difference. Allowing for a 10% rate of dropout rate, the sample size of a total of 120 patients was required.

### Statistical analyses

Continuous variables with normal and non-normal distribution are presented as the mean ± standard deviation and median (interquartile range), respectively. Differences of continuous variables among groups were analyzed using Student’s t-test, Mann Whitney U test, one-way ANOVA, and Kruskal–Wallis H test. All post hoc tests were Bonferroni correction. Categorical variables are presented as number (percentage) and compared with the Chi-square test. For analysis of the mean arterial pressure, heart rate and BIS value, repeated-measures ANOVA with Bonferroni correction was used. All data were processed by IBM SPSS Statistics 26.0 (SPSS Inc., Chicago, IL, USA). A 2-sided *P* value less than 0.05 was considered statistically significant.

## Results

### Basic clinical information of participants

**Fig. 1:**
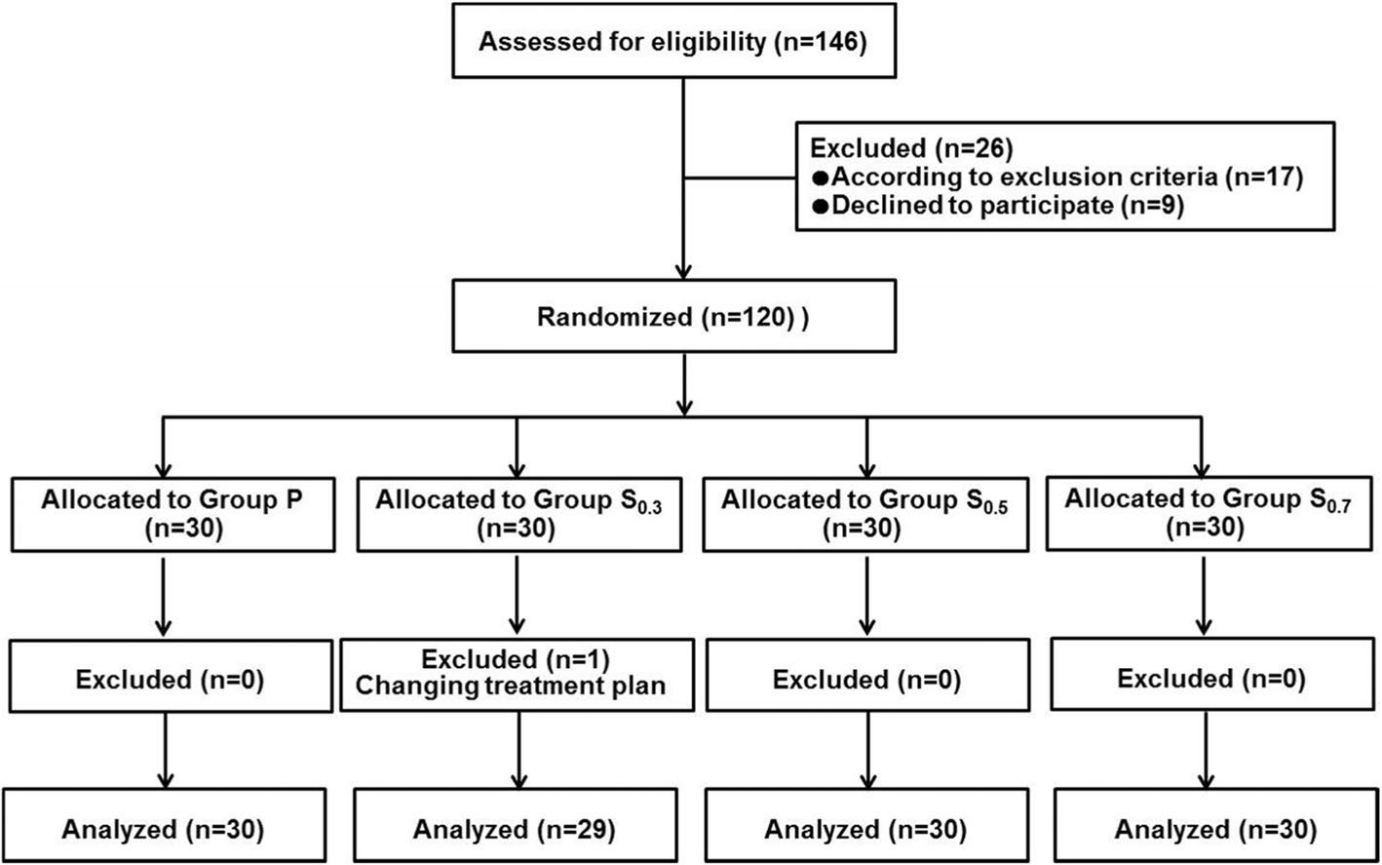
Flow diagram of participant enrollment

Initially, 146 eligible patients were screened. Finally, 120 patients were enrolled in this trial. One participant in Group S_0.3_ was excluded from analysis, and data from the remaining 119 participants were analyzed (Fig. 1). There were no significant differences in terms of baseline characteristics and procedure time among the groups (Table 1).

**Table 1:** Patient characteristics

	Group P (*n* = 30)	Group S_0.3_ (*n* = 29)	Group S_0.5_ (*n* = 30)	Group S_0.7_ (*n* = 30)	*P* value
Age (yr)	9.41 ± 2.06	9.92 ± 1.87	8.93 ± 1.95	9.45 ± 1.66	0.266
Sex (F/M)	17/13	12/17	13/17	14/16	0.647
Weight (kg)	34.70 ± 12.10	35.72 ± 10.85	34.12 ± 10.32	35.52 ± 10.21	0.937
Height (cm)	139.97 ± 15.89	138.62 ± 11.58	137.47 ± 14.10	137.67 ± 8.75	0.871
BMI (kg.m^−2^)	17.10 ± 2.80	18.13 ± 3.50	17.40 ± 3.02	18.37 ± 3.75	0.403
Procedure duration (min)	13.97 ± 3.38	12.55 ± 3.86	12.47 ± 2.32	13.07 ± 3.48	0.279

Data are expressed as mean (SD), median (interquartile range), or n, as appropriate

*yr* Year, *F* Female, *M* Male, *BMI* Body Mass Index

### Primary and secondary outcomes

Totally, there were 5, 10, 15, and 25 cases with smooth placement of first endoscope insertion in the in the Group P, Group S_0.3_, Group S_0.5_ and Group S_0.7_, respectively. The smooth placement rate of first endoscope insertion of Group S_0.3_, S_0.5_ and S_0.7_ were 34.50% (95%CI: 17.94%-54.33%), 50.00% (95%CI: 31.30%-68.70%) and 83.30% (95%CI: 65.28%-94.36%), respectively, which were significantly higher than that of Group P (16.70%, 95%CI: 5.64%-34.72%) (all *P* < 0.05). Group S_0.7_ had significantly higher smooth placement rate of first endoscope insertion than Group S_0.3_ and S_0.5_ (all *P* < 0.05). There was no significant difference in smooth placement rate of first endoscope insertion between Group S_0.3_ and Group S_0.5_ (Table 2).

**Table 2:** Sedation related conditions

	Group P (*n* = 30)	Group S_0.3_ (*n* = 29)	Group S_0.5_ (*n* = 30)	Group S_0.7_ (*n* = 30)	*P* value
Number of cases with smooth placement of first endoscope insertion	5	10	15	25	-
Smooth placement rate of first endoscope insertion (%)	16.70 (5.64 -34.72)	34.50 (17.94 -54.33)^†^	50.00 (31.30 -68.70)^†^	83.30 (65.28 -94.36)^†‡SS^	< 0.001
Times of additional propofol	2.50 (1.00–3.00)	2.00 (1.00–2.00)^†^	2.00 (1.00–2.00)^†^	1.00 (0.00–1.25)^†^	< 0.001
Total amount of propofol (mg.kg^−1^)	5.66 (4.28–6.97)	4.32 (3.87–4.94)	4.41 (3.92–4.88)	3.94 (3.00–4.30)^†^	0.003
Recovery time (min)	28.70 ± 15.66	29.45 ± 11.40	33.50 ± 16.47	35.67 ± 19.04	0.280
PACU stay (min)	45.27 ± 16.21	57.55 ± 15.59	58.53 ± 24.07	60.63 ± 23.74^†^	0.018
Endoscopist satisfaction rate	7.00 (6.00–7.00)	7.00 (7.00–8.50)	8.00 (7.00–10.00)^†^	9.00 (8.00–9.00)^†‡^	< 0.001

Data are expressed as rate (95% confidence interval), median (interquartile range) or mean ± standard deviation, as appropriate

*PACU* Post anesthesia care unit

^†^*P* < 0.05 vs. Group P; ^‡^ *P* < 0.05 vs. Group S_0.3_. ^SS^ *P* < 0.05 vs. Group S_0.5_

The times [median (interquartile range)] of additional propofol administration in Group S_0.3_, Group S_0.5_ and Group S_0.7_ were significantly less than that of Group P (Group P vs. Group S_0.3_, *P* = 0.018; Group P vs. Group S_0.5_, *P* = 0.014; Group P vs. Group S_0.7_, *P* = 0.001) (Table 2). Interestingly, although S-ketamine administration reduced the times of additional propofol administration, only Group S_0.7_ had significantly less total dose of propofol than Group P (3.94 vs 5.66 mg.kg^−1^, *P* = 0.003) (Table 2).

There was no significant difference in recovery time among the groups (*P* = 0.280, Table 2). However, length of PACU stay in Group S_0.7_ was significantly longer than that of Group P (*P* = 0.018). All groups had endoscopist satisfaction scores of at least 7 points, achieving satisfaction. However, the endoscopist satisfaction rate in Group S_0.7_ was significantly higher than that of Group P and Group S_0.3_ (Group P vs Group S_0.7_, *P* < 0.001; Group S_0.3_ vs Group S_0.7_, *P* = 0.001) (Table 2).

### Analysis of hemodynamic parameters and BIS value

As shown in Table 3, the heart rate at 1 min after induction in both Group P and Group S_0.3_ was significantly lower than that before induction (Group P: T_0_ vs T_1_, *P* = 0.014; Group S_0.3_: T_0_ vs T_1_, *P* = 0.047). The heart rate at 5 min during the procedure in Group S_0.3_ was significantly higher than that at 1 min after induction (Group S_0.3_: T_1_ vs T_5_, *P* = 0.005). The mean arterial pressure at 1 min after induction and at 10 min during the procedure in both Group P and Group S_0.3_ was significantly lower than that before induction (Group P: T_0_ vs T_1_, *P* < 0.001; T_0_ vs T_10_, *P* = 0.011. Group S_0.3_: T_0_ vs T_1_, *P* = 0.002; T_0_ vs T_10_, *P* = 0.001). However, there was no significant difference in mean arterial pressure of Group S_0.5_ and Group S_0.7_ among different points (Table 3).

**Table 3:** Analysis of hemodynamic parameters and BIS value

	Group P (*n* = 30)	Group S_0.3_ (*n* = 29)	Group S_0.5_ (*n* = 30)	Group S_0.7_ (*n* = 30)
**Heart rate (beats min**^**−1**^**)**				
T_0_: Before induction	92.15 ± 16.60	91.72 ± 16.64	90.71 ± 13.23	91.6 ± 12.35
T_1_: 1 min after induction	84.41 ± 11.78*	83.5 ± 14.16*	92.62 ± 13.47	89.84 ± 14.70
T_5_: 5 min during the procedure	86.33 ± 14.10	92.89 ± 15.68^†^	93.81 ± 17.42	88.56 ± 11.99
T_10_: 10 min during the procedure	87.59 ± 15.78	87.72 ± 14.37	89.67 ± 14.65	87.96 ± 13.95
**Mean artery pressure (mmHg)**				
T_0_: Before induction	77.70 ± 10.85	79.39 ± 8.85	79.19 ± 8.73	76.56 ± 8.41
T_1_: 1 min after induction	68.22 ± 7.21*	70.89 ± 9.24*	74.86 ± 6.00 ^SS^	72.88 ± 6.72
T_5_: 5 min during the procedure	69.96 ± 12.39*	78.39 ± 11.84^†^	77.52 ± 9.30	71.92 ± 7.55
T_10_: 10 min during the procedure	69 ± 10.84*	66.39 ± 16.96*^‡^	75.57 ± 10.29	70.8 ± 6.16
**BIS value**				
T_1_: 1 min after induction	55.00 ± 15.04	61.59 ± 15.19	58.19 ± 12.54	66.40 ± 16.38^SS^
T_5:_ 5 min during the procedure	58.54 ± 12.99	74.06 ± 10.58^†SS^	68.48 ± 10.39^†SS^	74.52 ± 10.18^†SS^
T_10_: 10 min during the procedure	60.69 ± 13.38	69.06 ± 10.74	68.9 ± 11.12^†^	71.48 ± 11.54^SS^

Data are expressed as mean ± standard deviation

*BIS* Bispectral index

^*^P < 0.05 vs. Before induction. ^†^*P* < 0.05 vs. 1 min after induction. ^‡^*P* < 0.05 vs. 5 min during the procedure. ^SS^*P* < 0.05 vs. Group P

At 1 min after induction, the BIS value of Group S_0.7_ was significantly higher than that of Group P (Group P vs Group S_0.7,_ *P* = 0.047), while the BIS value of Group S_0.3_ and Group S_0.5_ was not significantly different from that of Group P (both *P* = 1.000) (Table 3). At 5 min during the procedure, the BIS values of Group S_0.3_, Group S_0.5_ and Group S_0.7_ were significantly higher than those of Group P (Group P vs Group S_0.3,_ *P* < 0.001; Group P vs Group S_0.5,_ *P* = 0.020; Group P vs Group S_0.7,_ *P* < 0.001) (Table 3).

### Analysis of adverse events

Hypoxemia was the main adverse reaction caused by respiratory depression during induction, which was relieved by jaw thrust or mask ventilation (Table 4). The incidence of adverse reactions during the procedure in Group P and Group S_0.3_ was significantly higher than that in Group S_0.7_ (40.0%, 41.4% vs. 10.0%, *P* = 0.026). The high incidence of hypotension was the main contributor for the high overall adverse effects in Group P and Group S_0.3_. The incidence of hypotension in Group P and Group S_0.3_ was 23.30% and 24.10%, respectively, which was significantly higher than that in Group S_0.5_ and Group S_0.7_ (*P* = 0.01) (Table 4). All episodes were self-limited and did not require intervention. Dizziness was the most common adverse event in each group after surgery. The incidence of dizziness in Group S_0.7_ was higher than that of Group P (73.30% vs 26.70%, *P* = 0.003) (Table 4). Dizziness and visual impairment were both self-limited and did not require intervention.

**Table 4:** Analysis of adverse events

	Group P (*n* = 30)	Group S_0.3_ (*n* = 29)	Group S_0.5_ (*n* = 30)	Group S_0.7_ (*n* = 30)	*P* value
**After induction**					
Total number of adverse events	2 (6.7%)	2 (6.9%)	3 (10.0%)	3 (10.0%)	1.000
Akinetic state	0 (0.0%)	0 (0.0%)	0 (0.0%)	1 (3.3%)	1.000
Hypoxemia	2 (6.7%)	2 (6.9%)	3 (10.0%)	3 (10.0%)	1.000
**During the procedure**					
Total number of adverse events	12 (40.0%)	12 (41.4%)	8 (26.7%)	3 (10.0%)^† ‡^	0.026
Hypotension	7 (23.3%)	7 (24.1%)	1 (3.3%)^† ‡^	1 (3.3%)^† ‡^	0.01
Coughing/hiccups	5 (16.7%)	5 (17.2%)	6 (20.0%)	2 (6.7%)	0.503
Hypoxemia	2 (6.7%)	1 (3.4%)	1 (3.3%)	0 (0.0%)	0.753
**After the procedure**					
Total case of adverse events	10 (33.3%)	17 (58.6%)	18 (60.0%)	22 (73.3%) ^†^	0.016
Dizziness	8 (26.7%)	12 (41.4%)	13 (43.3%)	22 (73.3%)^†^	0.003
Visual disturbance	2 (6.7%)	7 (24.1%)	7 (23.3%)	5 (16.7%)	0.276
Headache	3 (10.0%)	1 (3.4%)	1 (3.3%)	3 (10.0%)	0.628
Nausea/vomiting	1 (3.3%)	0 (0.0%)	0 (0.0%)	1 (3.3%)	1.000
Fatigue	0 (0.0%)	1 (3.4%)	0 (0.0%)	0 (0.0%)	0.244
Abdominal pain	1 (3.3%)	0 (0.0%)	1 (3.3%)	0 (0.0%)	1.000

Data are expressed as n (%)

^†^ *P* < 0.05 vs Group P; ^‡^ *P* < 0.05 vs Group S0.3

## Discussion

In this study, we evaluated the sedative effects of S-ketamine in combination with propofol in school-aged children undergoing gastro-duodenoscopy. The results showed that S-ketamine could improve the tolerance and the smooth placement rate during endoscope insertion, which was positively related to the dosage of S-ketamine. In addition, the effects of different doses of S-ketamine on the intraoperative hemodynamics, the dosage of propofol, the adverse reactions after recovery and the length of PACU stay were different.

Endoscope insertion is a relatively difficult procedure in gastro-duodenoscopy and can cause severe stimulation. Some studies have used different methods or different drugs to reduce the stimulation during endoscope insertion and improve the satisfaction of the first insertion of the endoscope (Kramer, et al., [15], Gotoda, et al., [11]). Although propofol is the most common sedative for gastrointestinal endoscopy in children (Alletag, et al., [3]), it has no analgesic effect and requires adjuvant analgesia to reduce the stress response of children during gastro-duodenoscopy (Yan, et al., [24]). In this study, only 16.70% of the children who received propofol alone had smooth placement of first endoscope insertion. The smooth placement of the first time of endoscope insertion in children received 0.3 mg.kg^−1^, 0.5 mg.kg^−1^ and 0.7 mg.kg^−1^ was 34.5%, 50.00% and 83.3%, respectively, which was significantly higher than that of children who received propofol alone. This indicates that after induction with S-ketamine, the tolerance of the children may be increased, and that the smooth placement rate of the first time of endoscope insertion is significantly improved with the increase of the S-ketamine dose. It has been shown that ketamine can increase the sensitivity of the pharynx in children (Flores-Gonzalez, et al., [9]). In this study, administration of S-ketamine did not increase the incidence of pharyngeal sensitive symptoms (such as coughing and vomiting) during endoscope insertion. Importantly, tolerance to endoscopy was increased with increasing doses of S-ketamine. Therefore, we suppose that S-ketamine may have reliable analgesic and sedative effect, which could improve the pain tolerance and anesthesia depth and serve as the main contributor to the improvement in the smooth placement rate of endoscope insertion.

Some studies have shown that propofol in combination with other adjuvants (fentanyl, dexmedetomidine, ketamine, etc.) can significantly reduce the dosage of propofol during sedation for gastroscopy in children (Akbulut, et al., [2], Mason, et al., [16]), but some other studies yield different results. For example, Akbulut et al. showed that sedation with midazolam and propofol did not reduce the dose of propofol during gastroscopy in children (Akbulut, et al., [1]). In this study, there was no obvious difference in term of total dose of propofol between 0.3 or 0.5 mg.kg^−1^ S-ketamine and propofol alone. The total dose of propofol was only significantly reduced in children receiving 0.7 mg.kg^−1^ S-ketamine compared with propofol alone. Therefore, we suppose that when propofol is used in combination with other adjuvants, the dose of adjuvant is an important factor in determining the total amount of propofol. Similar results were also obtained in previous studies (Hayes, et al., [13], Zheng, et al., [26]). Hayes et al. showed that when the combination of ketamine and propofol were used for gastro-duodenoscopy in children, the dose of ketamine greater than 0.5 mg.kg^−1^ could reduce the total amount of propofol, but not the dose of 0.25 mg.kg^−1^. Zheng et al. reported that the total amount of propofol administered during sedation in combination with S-ketamine 0.5 mg.kg^−1^ and 1.0 mg.kg^−1^ was significantly less than those in combination with S-ketamine 0.25 mg.kg^−1^ (Zheng, et al., [26])..

BIS monitoring can be used to avoid the risk of excessive sedation and reduce the occurrence of adverse reactions and is recommended for endoscopy under propofol-based sedation (Gotoda, et al., [11]). However, it has been shown that intravenous ketamine under general anesthesia can affect the BIS value of children in a dose-dependent manner (Peltoniemi, et al., [19]). The 0.5 mg.kg^−1^ of ketamine can increase the BIS value but not 0.2 mg.kg^−1^. In adults receiving gastroscopy, it has also been shown that the BIS value in patients receiving propofol combined with ketamine was significantly higher than that in patients receiving propofol combined with dexmedetomidine (Tekeli, et al., [21]). Similar results were also obtained in this study. BIS value at 1 min after induction in children who received S-ketamine 0.7 mg.kg^−1^ was higher than that of children who received propofol alone. At 5 min during the procedure, the mean BIS values of children who received 0.3 and 0.5 mg.kg^−1^ S-ketamine were significantly higher than those of children who received propofol alone. This may be related to the fact that the application of S-ketamine reduced the number of additional intraoperative propofol. Thus, it is limited to use BIS monitoring alone during sedation with S-ketamine. The reactions of patients, such as heart rate fluctuations and physical movement, should also be monitored.

S-ketamine administration can increase sympathetic tone and decrease the risk of cardiorespiratory depression, which is the reason for the combined used of S-ketamine and propofol (Eich, et al., [7], Eberl, et al., [6]). In this study, children received 0.5 and 0.7 mg.kg^−1^ S-ketamine had no significant fluctuation in heart rate, and had a significant lower incidence of hypotension than that of children received propofol alone and 0.3 mg.kg^−1^ S-ketamine, which is suggestive of the properties of S-ketamine to maintain stable hemodynamics. In addition, the relatively small amount of propofol during the procedure was also the reason for the lower incidence of hypotension in these two groups. Zheng et al. showed that S-ketamine (0.5 mg.kg^−1^ and 1.0 mg.kg^−1^) combined with propofol for sedation reduced the total amount of propofol, and thus decreased the risk of propofol-related hemodynamic change (Zheng, et al., [26]). It has been confirmed that, in adults receiving gastroscopy, S-ketamine 0.5 mg.kg^−1^ could reduce the median effective concentration of propofol by 50%, and maintain a more stable hemodynamics (Yang, et al., [25]). Moreover, the effect of propofol on blood pressure is related to dosage (Yan, et al., [24]). In this study, the incidence of hypotension in children receiving propofol alone was 23.30%, which was similar to the findings of Narula et al. (Narula, et al., [18]). However, administration of 0.3 mg.kg^−1^ S-ketamine did not significantly decrease the incidence of hypotension (24.10%), suggesting that this dose of S-ketamine has a poor ability to excite sympathetic activity. It is necessary to note that although S-ketamine has less effect on the central respiratory drive, it can also produce respiratory depression when administered at high doses or rapidly (Trimmel, et al., [22]). In this study, the administration rate of S-ketamine and propofol was slowed down during induction, and the breathing rhythm of the children was always monitored. This is also the reason for the lower incidence of respiratory depression in this study even when S-ketamine was used in combination with a relatively high dose of propofol (3 mg.kg^−1^).

Akubulut et al. (Akbulut, et al., [2]) showed that dizziness (84.9%) and visual disturbance (74.1%) were the most common adverse events in children underwent gastro-duodenoscopy with sedation of ketamine combined with midazolam. In this study, the incidence of dizziness and visual disturbance was also higher than other adverse events. The incidence of dizziness was increased with increased doses of S-ketamine, which may be indicative of a dose-related side effect (Wang, et al., [23]). Psychotomimetic effects are also the concern following ketamine administration (Erstad and Patanwala, [8], Jalili, et al., [14]). No delirium and hallucinations was reported in this study, which may be related to the lower incidence of psychotropic adverse actions following S-ketamine administration and psychotic symptom inhibition of propofol (Trimmel, et al., [22], Friedberg, [10]).

Several studies have shown that propofol in combination with ketamine/S-ketamine can reduce recovery time in children (Eich, et al., [7], Harun, et al., [12]). However, in this study, the combination of propofol and S-ketamine did not show the advantage of rapid recovery. On the contrary, the recovery time of the children was prolonged with the increase of the dose of S-ketamine, although there was no statistical difference. In addition, 0.7 mg.kg^−1^ S-ketamine administration had increased length of PACU stay. The high incidence of dizziness (73.3%) may prolong the length of PACU stay.

There were several limitations in this study. First, we did not evaluate the doses of propofol required to suppress the stress response to endoscopic insertion in combination of different doses of S-ketamine. Therefore, when induction is performed with the combination of propofol and S-ketamine, the median effective dose of propofol for inhibiting the stimulation of endoscopic insertion still requires further study. Second, we did not observe the sedative effect of higher doses of S-ketamine in combination with propofol, and perhaps higher doses of S-ketamine could provide more satisfactory sedative effect and less total propofol dosage. However, side effects of S-ketamine are dose-related. High incidence of dizziness associated with S-ketamine (0.7 mg.kg^−1^) may suggest that higher doses of S-ketamine may have higher incidence of adverse effects.

## Conclusion

In summary, combined administration of S-ketamine and propofol can increase the tolerance of school-aged children during endoscopic insertion. Moreover, the smooth placement rate during the first endoscope insertion is positively correlated with the dose of S-ketamine. S-ketamine administration at 0.7 mg.kg^−1^ can maintain hemodynamic stability in children, reduce the number of additional propofol and the total amount of propofol, and improve endoscopist satisfaction. However, dizziness is the most common adverse event with 73.3% incidence and may prolong PACU stay.

## Data Availability

The datasets generated and/or analysed during the current study are not publicly available due to local ownership of the data but are available from the corresponding author on reasonable request.

## Abbreviations

BIS: Bispectral index
PACU: Post anesthesia care unit
